# DRD4 and the Impulsive Mind: Genetic Roots of Exploration and Risk

**DOI:** 10.1192/j.eurpsy.2026.11262

**Published:** 2026-07-31

**Authors:** A. A. Rehman, D. Valle, R. A. Maldonado-Puebla, B. R. Carr

**Affiliations:** University of Florida, Gainesville, FL, United States

## Abstract

**Introduction:**

The dopamine D4 receptor gene (DRD4), particularly its 7-repeat (7R) variable number tandem repeat (VNTR) in exon III, is linked to novelty-seeking, impulsivity, and risk-taking behaviors. This allele, which shows reduced dopaminergic signaling, is overrepresented in migratory populations, suggesting an adaptive role in promoting exploration. Clinically, the 7R variant is associated with attention-deficit/hyperactivity disorder (ADHD), substance use vulnerability, and other externalizing disorders. DRD4 also influences executive function and behavioral regulation via neural circuits involved in reward sensitivity and cognitive control.

**Objectives:**

This poster reviews the behavioral, neurodevelopmental, and clinical significance of the DRD4 7R allele. We propose a framework in which this variant modulates susceptibility to impulsivity and risk-related behaviors through interactions among dopaminergic signaling, environmental exposures, and executive processes.

**Methods:**

We conducted a literature review using PubMed, PMC, and Google Scholar, focusing on studies from the past two decades examining the behavioral and neurocognitive effects of the DRD4 7R polymorphism. Articles were included if they addressed dopaminergic signaling, executive function, ADHD, externalizing behaviors, or environmental modulation of genetic risk. Priority was given to peer-reviewed studies integrating neuroimaging, developmental psychopathology, and behavioral genetics.

**Results:**

The DRD4 7R allele is associated with greater risk for externalizing behaviors such as sensation-seeking, early substance use, gambling, and ADHD symptoms. Mechanistically, it encodes a receptor with diminished intracellular signaling, which may prompt compensatory behaviors that enhance dopaminergic activity. Neurobiologically, DRD4 is expressed in the prefrontal cortex and influences reward sensitivity, inhibitory control, and executive functioning through the mesocorticolimbic pathway. The 7R variant has also been linked to impaired prefrontal signaling and altered NMDA receptor activity, contributing to impulsivity. Gene-environment interactions are central to its effects: adverse conditions like inconsistent parenting, prenatal stress, or early adversity increase externalizing risk, while supportive environments can buffer against it. Evidence also suggests potential modulation of stimulant response in ADHD, though findings are mixed.

**Conclusions:**

The DRD4 7R allele acts as a genetic modulator of behavioral development, shaping impulsivity and externalizing risk through dopaminergic signaling, executive function, and environmental sensitivity. It functions less as a fixed risk factor than as a context-sensitive amplifier, heightening both maladaptive and adaptive trajectories. This developmental framework highlights the importance of integrating genetic predispositions with psychosocial factors to guide personalized prevention and intervention strategies.

**Disclosure of Interest:**

None Declared

